# Effect of Various LED Light Qualities, Including Wide Red Spectrum-LED, on the Growth and Quality of Mini Red Romaine Lettuce (cv. Breen)

**DOI:** 10.3390/plants12102056

**Published:** 2023-05-22

**Authors:** Joo Hwan Lee, Yong Beom Kwon, Yoo Han Roh, In-Lee Choi, Jidong Kim, Yongduk Kim, Hyuk Sung Yoon, Ho-Min Kang

**Affiliations:** 1 Interdisciplinary Program in Smart Agriculture, Kangwon National University, Chuncheon 24341, Republic of Korea; ot2581@naver.com (J.H.L.); nm96727@naver.com (Y.B.K.); nuh3722@naver.com (Y.H.R.); 2 Agricultural and Life Science Research Institute, Kangwon National University, Chuncheon 24341, Republic of Korea; cil1012@kangwon.ac.kr; 3 FutureGreen Co., Ltd., Yongin 17095, Republic of Korea; jidong.kim@futuregreen.co.kr; 4 Cheorwon Plasma Research Institute, Cheorwon 24062, Republic of Korea; ydkim@cpri.re.kr; 5 The Waksman Institute of Microbiology, Rutgers the State University of New Jersey, Piscataway, NJ 08854, USA

**Keywords:** led, light quality, mini red romaine lettuce, anthocyanin, bitterness

## Abstract

Recently, LEDs with various light qualities have been used in closed plant factories, and they are known to have different effects on the growth and quality of crops. Therefore, this study was conducted to investigate the change in growth and quality in mini red romaine lettuce using LEDs with various light qualities. Wide red spectrum (WRS)-LEDs, blue (B)-LEDs, blue + red (BR)-LEDs, red (R)-LEDs, and white (W)-LEDs were used as the artificial light sources. Regarding growth, the R-LED treatment showed the most positive effect, but the leaf shape was not normal and the Hunter b* value was not suitable because it was higher than that of the other treatments. The Hunter a*, SPAD, and NDVI values of the B- and BR-LED treatments were effective, but this was not the case for those of the R- and W-LED treatments. The anthocyanin reflectance index 1 (ARI1) was 20 times higher in the B-LED treatment than in the R-LED treatment, and the ascorbic acid content was the highest in the WRS-LED treatment. In the sensory evaluation, bitterness and sweetness showed opposite tendencies. Regarding the overall preference, the BR-LED treatment received the highest score. Correlation analysis showed that the bitterness was closely correlated with the anthocyanin content and leaf color. Taken together, BR-LEDs provided a good top fresh weight, dark red leaves, and high anthocyanin and ascorbic acid contents, with the highest overall preference; therefore, BR-LEDs were the most suitable for the cultivation of mini red romaine lettuce.

## 1. Introduction

According to the Food and Agriculture Organization, more than one million hectares of lettuce are cultivated worldwide, with a production of more than 22 million tons in 2022 [1]. Lettuce, which is mainly consumed as a fresh-cut salad, can provide phenolic compounds, flavonoids, carotenoids, and vitamin C, and is consumed worldwide, as it can be produced year-round [2]. In particular, red romaine lettuce, which has a red leaf color, can play a beneficial role in health by accumulating a large amount of anthocyanin, which is well known for its antioxidant action, in its tissues [3]. One of the major characteristics of lettuce is its bitter taste, and lactucopicrin, which is among the major bitter sesquiterpene lactones (BSLs), was reported to be the main cause [4]. In addition, BSLs can act as an important factor in consumer purchasing, and if the content is too high, preference can be significantly reduced [5,6].

Currently, LEDs (light-emitting diodes) are the main light source among artificial light sources used in plant factories [7]. LEDs have various advantages over existing artificial light sources, such as a long lifetime, higher electrical conversion efficiency, and lower heat generation and price [8,9]. However, the most important factor in crop cultivation is that they can produce numerous types of light quality (spectrum) by combining different wavelengths, providing the optimal light quality according to the crop type and growth stage [10,11]. It is reported that these different combinations of LEDs can have various effects on the growth and quality of crops [7,8].

In plants, the main photosynthetic pigments, namely, chlorophyll *a* and *b*, absorb most of the blue and red light in the range of 400–700 nm, which is known as photosynthetically active radiation (PAR). Additionally, they can respond through photoreceptors, such as cryptochrome, which detects blue light, and phytochrome, which detects red light [11]. Previous studies also reported that the combination of blue and red light is the most effective for the growth and development of many leafy vegetables, including lettuce [12]. Based on the above reasons, blue (B)-, red (R)-, blue + red (BR)-, white (W)-, and wide red spectrum (WRS)-LEDs were used as artificial light sources in this study.

Blue light emitted from B-LEDs (400–500 nm) is known to act as an important wavelength for the formation of biomass, anthocyanin, chloroplasts and chlorophyll, and photomorphogenesis in lettuce and various crops [13,14], but they have the disadvantages of producing a small leaf size and a slow growth rate when used alone at a high light intensity [15,16]. Conversely, R-LEDs (600–700 nm) act as the most effective wavelength for the growth rate and photosynthesis of lettuce in plant factories, but it reduces the phenol content and chlorophyll relative to B-LED [11,17].

Artificial light sources widely used as mixed light include BR-LEDs and W-LEDs. BR-LEDs (400–500 + 600–700 nm) were reported to improve the accumulation of phenolic compounds and growth through a synergistic effect when irradiated with a mixture of B- and R-LEDs compared with when B- and R-LEDs were used alone [18,19]. In addition, Kang et al. [15] reported that BR(2:8)-LEDs provided the greatest increase in the photosynthetic rate of lettuce compared with B- and R-LEDs alone. Unlike BR-LEDs, W-LEDs (400–700 nm) contain a large amount of green light in their spectrum. In a previous study, it was reported that the biomass and growth rate of lettuce increased when green light was added to a BR-LED [20,21]. However, it has also been found that the photosynthetic rate is greatly reduced without affecting the growth of lettuce [15].

WRS-LEDs are artificial light sources that use quantum dots, whose optical and electrical properties change when a semiconductor is reduced to nanometer (nm) size. They have a wider light distribution angle than conventional LEDs, and thus, their uniformity is high, and they have the advantage of being able to produce the Emerson synergy effect, which increases the photosynthesis rate compared with when other wavelengths are irradiated independently [22]. In this experiment, the wavelengths of WRS-LEDs included 26.5% blue (B) light (400–500 nm), 12.2% green (G) light (500–600 nm), 50.8% wide-red (R) light (600–700 nm), and 10.5% far-red (FR) light (700–800 nm). FR light has been mentioned as a necessity for plants to perform efficient photosynthesis and photochemistry [23], and it is sensed by phytochrome, together with red light, and is known to show higher leaf transmittance than red light [11]. In addition, it causes a shade avoidance reaction in plant growth, increases the size of leaves, and elongates stems, which can cause significant changes in plant morphology [24]. According to a previously reported study, when lettuce was treated by adding FR light to blue and red light, the biomass increased by 39% and 25%, respectively, and the appearance of the plant was changed to improve the light use efficiency [25]. In addition, Hwang et al. [26] reported that as a result of cultivating tomatoes, peppers, cucumbers, and watermelons by supplementing far-red rays with cool-white LEDs, the hypocotyl length and dry weight of seedlings increased as the light intensity of far-red rays increased. Furthermore, Tan et al. [27] reported far-red-induced changes in plant height, leaf structure and shape, stomatal response, chloroplast development, biomass, photosynthetic pigment and fluorescence, electron transport, carbon assimilation, etc., in various crops.

As explained in the papers referenced above, the growth and quality of plants can vary depending on the light quality, and among LEDs of various light qualities, BR-LED, which is a mixed light, was found to be effective in cultivation [8,15,18]. In particular, WRS-LED is expected to be more effective than existing artificial light sources by making up for the shortcomings of existing LEDs, utilizing a wide light distribution angle, a wide red spectrum, and far-red rays. Therefore, this study was conducted to identify the growth and quality changes in mini red romaine lettuce (cv. Breen) using LEDs with various light qualities in a closed plant factory-type chamber.

## 2. Results

**Figure 1:**
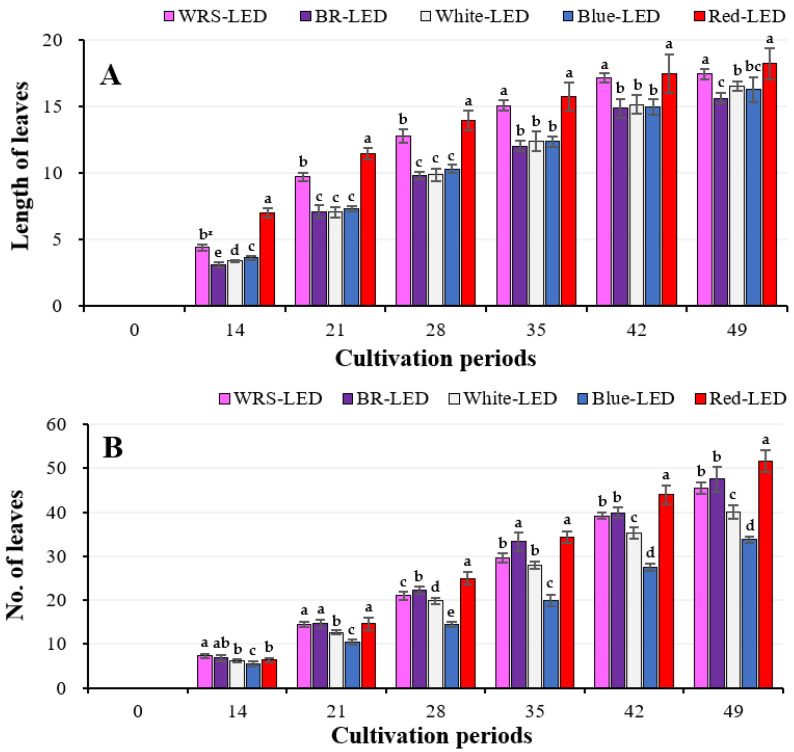
Change in the length of leaves (**A**) and the number of leaves (**B**) of mini red romaine lettuce cultivated under LEDs with various light qualities for 49 days. Vertical bars represent ± SD (n = 6–8). ^z^ Mean separation within columns by Duncan’s multiple range test at 5% level. Values marked with different letters indicate significant differences according to Duncan’s multiple range test at the 5% level.

During the entire cultivation period, lettuce grown under R-LEDs produced the longest leaf length compared with the other treatments, followed by the WRS-LEDs. The difference in leaf length of lettuce cultivated under R-LEDs and WRS-LEDs up to the 28th day showed statistical significance, with a difference of about 2 cm, but there was no difference from the 35th day (Figure 1A). The number of leaves showed a consistent trend until the 49th day, except for the 14th day of cultivation. At the end of cultivation, lettuce grown under R-LEDs produced the most number of leaves, with 51.7 leaves, showing a significant difference from the other treatments, followed by the BR-, WRS-, W-, and B-LED treatments in order. On the 49th day, lettuce grown under R-LEDs produced 35% more leaves than lettuce grown in B-LEDs, which produced the fewest leaves (Figure 1B).

At the end of cultivation, the top fresh weight was significantly the biggest in lettuce grown in the R-LED treatment among all treatments, producing the highest leaf length and the number of leaves. The top fresh weight of lettuce grown under the R-LED treatment on the 49th day was 25.7% bigger than that of the BR-LED treatment, which was the second biggest, and 43.5% bigger than that of the B-LED treatment, which was the lowest. In the R-LED treatment, which produced the biggest top fresh weight, the number of leaves and top fresh weight on day 49 showed the same trend. However, there was no statistical significance between the BR- and WRS-LEDs, or between the W- and B-LEDs (Table 1). The top dry matter ratio was the highest in lettuce cultivated under the BR-LED treatment, which produced the second-biggest top fresh weight, but there was no statistically significant difference from the rest of the treatments, except for the R-LED treatment. Lettuce grown in the R-LED treatment, which produced the biggest top fresh weight, showed the lowest top dry matter ratio, and conversely, the B-LED treatment, which produced the smallest top fresh weight, showed the second-highest top dry matter ratio (Table 1).

**Figure 2:**
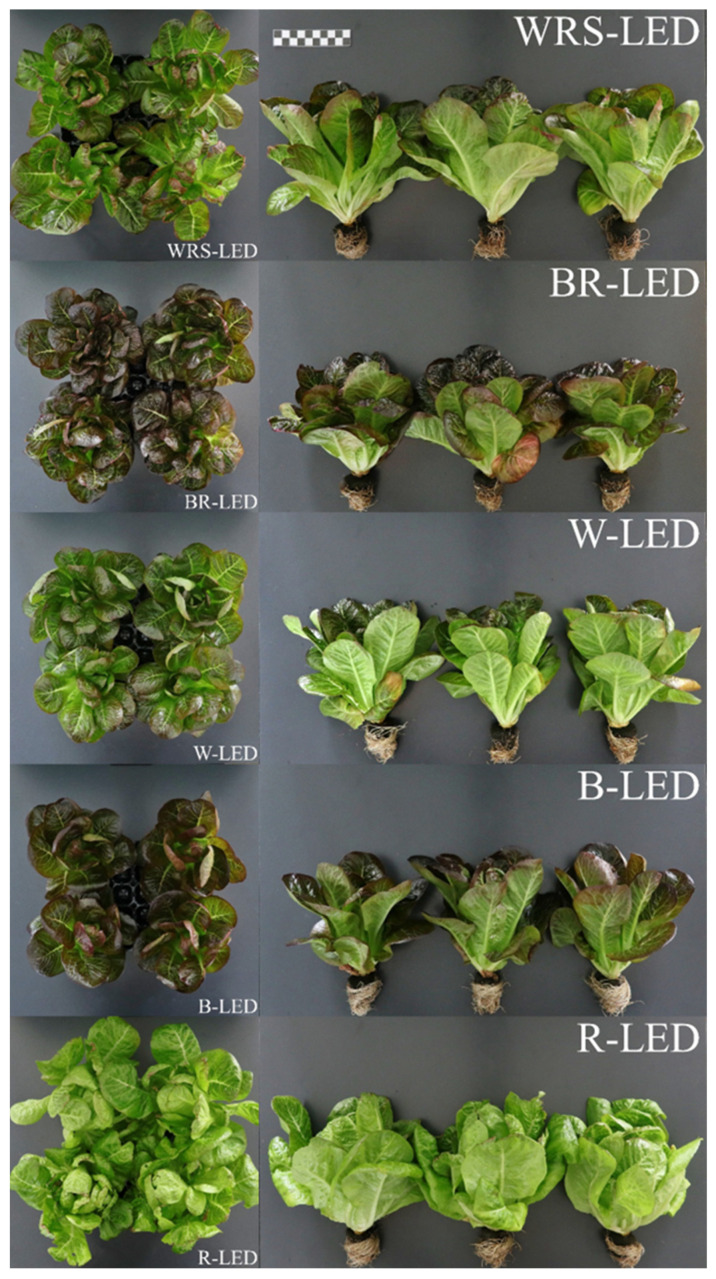
Mini red romaine lettuce cultivated for 49 days under LEDs with various light qualities.

As for chromaticity, Hunter L* (closer to 100, whiter; closer to 0, blacker) was the highest in the R-LED treatment, and lettuce grown under BR- and B-LEDs showed a lower value, resulting in a darker leaf color. Regarding Hunter a* (+ is redder and − is greener), lettuce grown in the B-LED treatment produced the deepest red color, and there was no significant difference from the BR-LED treatment, which had the second-highest value. Hunter a* values showed negative results with green leaves only in the R-LED treatment with the best growth, and all lettuce grown in other treatments produced red leaves, which can be visually confirmed in Figure 2. Regarding Hunter b* (+ is yellower and − is bluer), the B- and BR-LED treatments, which produced the deepest red color in lettuce leaves, had the lowest values without statistical significance. In contrast, lettuce grown in the R-LED treatment, which produced green leaves, showed a significantly higher value compared with the other treatments (Table 2).

The soil plant analysis development (SPAD), which can represent the chlorophyll content, was the highest in lettuce grown with the BR-LED treatment and was significantly higher than that of the R-LED treatment, which had the lowest chlorophyll content, by more than 24%. In addition, in lettuce cultivated in the BR-, B-, WRS-, W-, and R-LED treatments, the chlorophyll content showed the same trend as the top dry matter ratio, but the difference between the treatments was more pronounced in the chlorophyll content (Table 3). The normalized difference vegetation index (NDVI) is a value that is proportional to the chlorophyll change and plant health status. Similar to SPAD, it was the highest for lettuce grown under the B-LED and BR-LED treatments without significance, and the R-LED treatment showed the lowest value. In the Polypen manual for measuring the NDVI, the value ranges are stated as 0.5–0.9 for healthy leaves and 0.2–0.4 for unhealthy leaves. Only the lettuce cultivated under WRS-, BR-, and B-LED treatments had values corresponding to the range for healthy leaves, with values of 0.519, 0.530, and 0.550, showing statistical significance compared with the W- and R-LED treatments, which had values corresponding to the range for unhealthy leaves (Table 3). The anthocyanin reflectance index 1 (ARI1) reflects changes in the anthocyanin content. In this study, the trend of ARI1 was the same as that of Hunter a*. Lettuce grown under the B-LED treatment produced the highest anthocyanin content, which was more than 3 times higher than that of the BR-LED, WRS-LED, and W-LED treatment groups, and 20 times higher than that of the R-LED treatment group, which produced the lowest content. Summarizing the results of ARI1, it was shown that blue light increased the red color expression of red romaine lettuce, while red light and green light decreased it. In addition, in the case of Hunter a*, there was no significant difference between lettuce cultivated under the B- and BR-LED treatments, indicating that blue light was responded to more sensitively than red light to produce the red color expression of red romaine lettuce. However, ARI1 showed more than twice the difference between the B- and BR-LED treatments (Table 3). It seems that the degree of difference in the results between the treatment groups was due to the difference in the measurement method of Hunter a* and ARI1. The ascorbic acid content was the highest in lettuce grown under the WRS-LED treatment at 4.40 mg/100 g FW, which was 38% significantly higher than that under the R-LED treatment, which had the lowest value. The reason why the lettuce grown under the WRS-LED treatment was able to produce the highest ascorbic acid content is thought to be due to the FR light. However, it did not seem to have a significant effect, as there was no significant difference between the BR- and B-LED treatments. The red light treatment demonstrated a low ascorbic acid synthesis ability when used alone in lettuce, but when irradiated with blue light, the content was higher than that of blue light alone due to the positive synergy (Table 3). Under the W-LED treatment, which produced the lowest content among the mixed lights, the green light in the spectrum seemed to interfere with the ability of the blue light to synthesize ascorbic acid.

**Figure 3:**
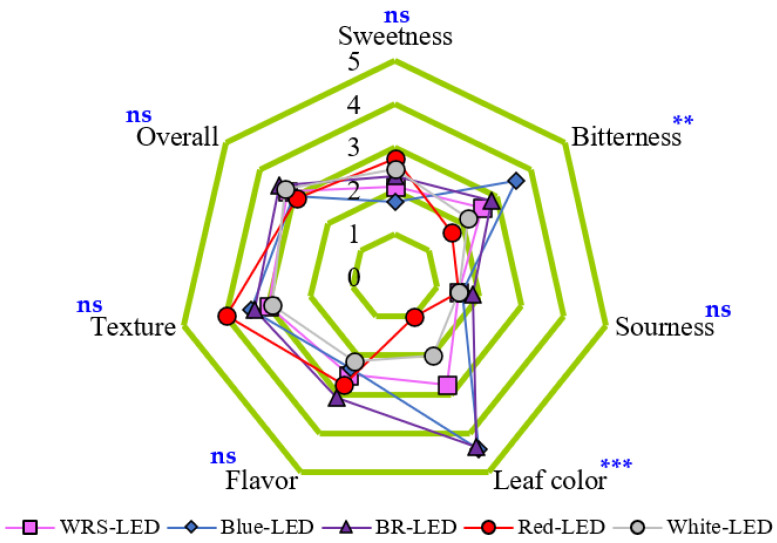
Sensory evaluation of mini red romaine lettuce cultivated under LEDs with various light qualities on the final day. ns, **, and *** indicate non-significant and significant differences at *p* < 0.01, and 0.001, respectively. Sweetness, bitterness, sourness, and flavor: 5 = very strong, 4 = strong, 3 = normal, 2 = faint, and 1 = very faint. Leaf color: 5 = very deep, 4 = deep, 3 = normal, 2 = light, and 1 = very light. Texture and overall preference: 5 = very good, 4 = good, 3 = normal, 2 = bad, and 1 = very bad.

The sweetness was the highest for lettuce grown under the R-LED treatment and the lowest for the B-LED treatment. Contrary to sweetness, bitterness was the highest for lettuce cultivated under B-LEDs and showed a stronger bitter taste than lettuce cultivated under R-LEDs, which was the lowest, by 53%. In addition, there was a clear difference between the treatment groups in bitterness rather than sweetness. The results of sweetness and bitterness were related to the Hunter a* and ARI1 results: sweetness was low and bitterness was high when the leaf color was deep red, and the opposite tendency was shown when the leaf color was green. However, it is known that a strong bitter taste can reduce consumers’ purchase preferences [5,6]; in this study, the overall preference for B-LED-treated lettuce, which had a red leaf color and the strongest bitter taste, was the second lowest among all treatments groups (Figure 3). Sourness was the highest for lettuce grown under BR-LEDs, but due to the nature of these crops, the sourness was investigated as low, i.e., less than 1–2 points, in all treatment groups; therefore, it did not seem to be affected by the light quality. Among the sensory evaluation items, the difference in leaf color was the greatest between the treatment groups, and the change due to the light quality was large. As for leaf color, which tended to show the same trend as Hunter a* and ARI1, the B-LED and BR-LED treatments, which produced dark red leaves, scored high, followed by the WRS-LED, W-LED, and R-LED treatments. The highest flavor score was obtained by lettuce cultivated under the BR-LED treatment, while the lowest was found for the W-LED treatment. However, since there were no similar or identical trends among the survey items investigated in this study, additional research on the flavor of lettuce according to the light quality is necessary. Texture obtained the highest preference score for lettuce grown under R-LEDs. The reason for this is not indicated in Figure 3, but the judges said that the leaf tissue was soft, and thus, gave it a higher score than the other treatments. Finally, for the overall preference, lettuce grown under the BR-LED treatment received the highest score, followed by the W-LED, WRS-LED, B-LED, and R-LED treatments, but there was no statistical significance (Figure 3).

**Figure 4:**
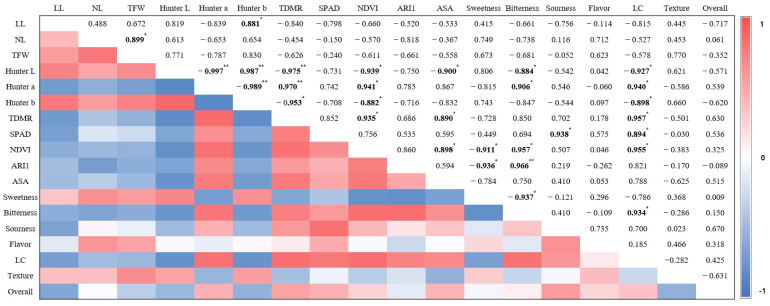
Correlation analysis of red romaine lettuce growth and quality characteristics when cultivated under LEDs with various light qualities on the final day. LL: leaf length; NL: number of leaves; TFW: top fresh weight; TDMR: top dry matter ratio; NDVI: normalized vegetation index; ARI1: anthocyanin reflectance index 1; ASA: ascorbic acid; LC: leaf color. * indicates a significant correlation at *p* < 0.05; ** indicates an extremely significant correlation at *p* < 0.01.

The growth and quality characteristics of mini red romaine lettuce were analyzed via correlation analysis, as shown in Figure 4. First, the top fresh weight was found to have a significant positive correlation with the number of leaves, with 0.899, indicating that the increase was due to the number of leaves rather than the length of the leaves. Hunter a* showed a high negative correlation with Hunter b* at the *p* < 0.01 level, and it increased statistically significantly with bitterness in the sensory evaluation. In addition, Hunter a*, the top dry matter ratio, the ascorbic acid content, and the bitterness had important effects on the NDVI, which can be used as an indicator of plant health. ARI1, which is proportional to the anthocyanin content, showed a negative correlation with sweetness and a positive correlation with bitterness, while sweetness and bitterness showed a negative correlation with each other at the 95% level. As a result, in this study, the plants with a dark red color, high top dry matter ratio, high ascorbic acid content, and strong bitter taste were healthy, whereas dark yellow leaves were unhealthy. Comparing the above results with the various LEDs used in this study, it can be said that lettuce grown under BR- and B-LEDs was healthy, but lettuce grown under R-LEDs was not. WRS-LEDs also produced most of the conditions for healthy lettuce, but could not produce all of them due to a low expression of leaf color. In addition, the sweetness and bitterness of the lettuce showed opposite tendencies, where the higher the anthocyanin content, the stronger the bitterness and the lower the sweetness (Figure 4).

## 3. Discussion

It is known that LEDs, which can be easily controlled according to the requirements of plants and can have various light qualities, can affect plant growth, biomass, and functional compounds in various ways [7,8].

Shimizu et al. [28] suggested that red light may be the most effective wavelength for photosynthesis and growth rates when growing lettuce in a plant factory. In addition, in a previous study, the number of leaves and the photosynthetic rate also showed the highest values under red light, followed by a BR-LED treatment [29]. This is the same trend as that found in the results of this study, which provides a basis for the results of the longest leaf length and the largest number of leaves found in lettuce grown under red light alone (Figure 1A,B).

The 49th-day top fresh weight also showed the highest result under the R-LED treatment (Table 1). In a previous study, lettuce grown under red light also showed the biggest top fresh weight, which was attributed to the high photosynthetic rate [28]. However, Chen et al. [30] reported that lettuce grown under red light grew rapidly, but when the ratio of red light was over 70%, the petiole distortion was evident, and with 100% red light, the original lettuce shape was lost. Similarly, in this study, it was difficult to see that the lettuce cultivated under R-LEDs was commercially viable, as it showed heterogeneity in the leaves and overall shape compared with lettuce grown under other light qualities (Figure 2). Therefore, it seems that lettuce cultivation under BR-LEDs, which produced the second biggest top fresh weight after R-LEDs, as well as a normal leaf shape, is more suitable. It has been reported that among LEDs of various light qualities used in this study, FR light, which is included only in WRS-LEDs, can significantly change photomorphogenesis by causing shade avoidance symptoms during plant growth [24]. With these characteristics, it has been stated that adding far infrared rays to the existing light source can reduce seedling size variation within the same cultivation bed [31]. For lettuce grown in the WRS-LED treatment, the standard deviation of the top fresh weights by wider light distribution angle, wide red spectrum, and FR rays was the smallest at 2.94, confirming that cultivation among individuals within the same treatment group proceeded uniformly (Table 1). However, as there was no difference from lettuce cultivated under the BR-LED treatment in terms of the top fresh weight, the wider light distribution angle, wide red spectrum, and FR rays seem to have produced a greater uniform cultivation and shade avoidance effect than growth enhancement. Lettuce grown under the B-LED and W-LED treatments showed poor growth compared with the other treatments (Table 1). This coincides with the results of Kang et al. [15], who found that blue light slowed down the growth rate of lettuce and that the green light comprising 30% of the W-LED did not have a positive effect on the growth rate. Previous papers reported that green light reduces the photosynthetic rate by reducing the chlorophyll content and stomatal conductance [32,33], but does not affect plant growth [15]. However, this does not mean that it does not affect lettuce growth at all, and it is thought that green light does not cause a direct growth reduction mechanism in lettuce, but indirectly affects the photosynthetic rate and stomatal conductance, leading to a growth reduction.

The degree of redness (Hunter a*) expressed in red romaine lettuce leaves is known to have a strong positive correlation with the actual anthocyanin content, with an R^2^ value of 0.80 [34]. Blue light, for which the Hunter a* value was the highest in this study (Table 2), is particularly used as an essential wavelength for anthocyanin synthesis in red lettuce [13]. However, it has been reported that R-, G-, and FR-LEDs do not synthesize anthocyanin in lettuce or suppress the effect of blue light to prevent the red expression of the leaves [13,35,36], and the same result was confirmed in this study (Table 2). In a previous study, R-LEDs with a high ratio of red light and R:FR did not detect that a leaf was under other leaves, and thus, the expression of SAG (senescence-associated gene) family genes (e.g., x SAG13) related to leaf senescence was suppressed [37]. However, in this study, the uniquely high Hunter b* value in lettuce grown under the R-LED treatment were significantly negatively correlated with the NDVI (Figure 4). This means that the higher the Hunter b* result, the poorer the health of the plant, and the increase in yellow color in lettuce, which usually has green or red leaves, mainly means yellowing of the leaves. Therefore, in this study, it seems that the yellowing of the leaves progressed relatively quickly in lettuce cultivated under the R-LED treatment compared with the other treatments. 

BR-LEDs are effective in promoting plant growth and biomass accumulation [8,15]. However, in this study, due to the characteristics of lettuce, there was no difference between the treatment groups, except for the R-LED group (Table 1), because the body water content was more than 95%. It was reported that FR light absorbs more water than BR light and increases the amount of water in the cell, which increases the expandability of each cell, thereby increasing the ratio of top fresh weight to top dry weight [38]. In addition, in previous studies, it was found that red light increased the top fresh weight but decreased the top dry weight [29] and that the top dry weight of red lettuce grown under R-LEDs was lower than that of lettuce grown under B-LEDs and BR-LEDs [35].

Blue light promoted 5-aminolevulinic acid (ALA), a precursor of chlorophyll tetrapyrrole, and suppressed the decrease in ALA caused by red light, resulting in the recovery of the chlorophyll concentration [39,40]. Zheng et al. [41] reported that lettuce grown in BR-LED, a mixed light, had a higher chlorophyll content than B- and R-LEDs alone due to a synergistic effect. Additionally, when irradiation was performed by adding FR light (50 μmol·m^−2^·s^−1^) to a BR-LED (200 μmol·m^−2^·s^−1^) for 16 h during the day and 1 h at the end of the day, the chlorophyll content of lettuce decreased [25]. Green light also downregulates transcription factors for chloroplast formation, reducing the chlorophyll content [42].

The NDVI is an indicator that can be used to check the health status, stress level, and chlorophyll concentration of plants by comparing the amount of red light, which is a part of visible light, with the amount of deflected NIR light. Alsina et al. [43] found the highest levels of chlorophyll *a* + *b* when ‘Lollo Bionda’ lettuce was grown under blue light, followed by BR light, with the lowest levels under R light. In addition, during lemon balm cultivation, W-LEDs containing some green light had a negative effect on the NDVI [44]. Both NDVI and SPAD are used as indicators of chlorophyll content, and in this study, the two trends were similar, but the difference in the degree of significant difference between treatments was considered to be due to the different measured infrared wavelength bands (Table 3). SPAD’s infrared wavelength band is measured at 940 nm, while that of the NDVI is measured in the 770–900 nm range.

The expression of genes that induce anthocyanin synthesis is induced by blue light and is known to be mediated by cryptochrome (Cry1) [45]. Cryptochrome, which absorbs and reacts with blue light treatment, has a total of two maximum absorptions at the 375 nm chromophore 5,10-methenyltetrahydrofolic acid (MTHF) and the 450 nm flavin adenine dinucleotide (FAD) chromophore flavin [46]. These flavin chromophores are reduced to half-forms under blue light, and cryptochromes become inactive under green and yellow light [42]. Light that can interfere with anthocyanin expression includes green light, red light, and far-red light. First, green light has an opposite tendency to blue light. In a previous study, when green and blue lights were used at the same time compared with when blue light was used alone, it was reported that in lettuce the oxidized flavin content was greatly reduced and the anthocyanin level was low [36]. In addition, it was reported that the completely reduced form of flavin showed the same movement as the flavin light balance in vivo caused by green light, and finally, the overall oxidized and reduced form of Cry1 was reduced [36]. In the case of red light, this can be explained by its effect on gene expression in the anthocyanin biosynthesis process. In previous studies, the gnl|UG|Lsa#S56341499 gene among lucoanthocyanidin oxidase (LDOX) coding genes and gnl|UG|Lsa#S58677322 gene among dihydroflavonol 4-reductase (DFR) coding genes were most frequently expressed under blue light during anthocyanin biosynthesis; however, they were not well expressed in lettuce grown under red light [37]. Finally, it was reported that in lettuce the anthocyanin concentration was reduced by up to 40% when FR light was also irradiated with white fluorescent light [13].

The assimilation of ascorbic acid in plants is significantly affected by light and temperature, and it is known that the light environment in particular has an important effect on the ascorbic acid metabolic pathway [47,48]. Chen et al. [49] reported that the ascorbic acid content in lettuce was higher when grown under B-LEDs and BR-LEDs compared with lettuce cultivated under R-LEDs. Adding green light to BR-LEDs resulted in a 44% reduced ascorbic acid content of lettuce compared with the use of BR-LEDs alone [50]. In addition, it was reported that W-LEDs supplemented with FR light increased the accumulation of ascorbic acid in green lettuce by 45% compared with W-LEDs alone, but they reduced the pigments and biomass [51]. However, as a result of adding red light to W-LEDs, there was no difference in the ascorbic acid content of lettuce compared with W-LEDs alone [51].

Even in recent studies, it is difficult to find content that sensory evaluation results for lettuce play an important role in deriving the final result, and most of them are comparisons of sensory evaluation parameters according to treatment groups [52,53,54]. In this study, there were some clearly distinguished results, such as bitterness and leaf color, but most did not show significant differences according to light quality. Therefore, this author believes that consumers’ sensory evaluation may not play a large role in determining the optimal light quality, but can be used as a reference. However, consumers’ sensory evaluation of lettuce can explain the difference for each parameter via comparison according to light quality. Meng et al. [54] reported that B_20_G_60_R_100_ had significantly higher sweetness than B_100_G_60_R_20_ as a result of the sensory evaluation of ‘Rouxai’ red oakleaf lettuce. Green light supplemented with oak lettuce was reported to be related to the activity or synthesis of enzymes related to sugar metabolism [51]. In this study, W-LEDs containing green light produced a higher sugar content than lettuce grown under BR-LEDs (Figure 3), but Nur Syafini et al. [55] reported that soluble solid contents (°Brix) were significantly reduced when green light was added to BR-LEDs. These conflicting results may be due to differences in the respective ratios of red, blue, and green light within the light source, or because the sweetness perceived by humans and the measured soluble solid contents (°Brix) are not proportional. It is known that the bitterness of lettuce is mainly affected by the content of the compounds lactucin, 8-deoxylactucin, and lactucopicrin, which are types of bitter sesquiterpene lactones (BSLs) [4]. In a previous study, the bitterness of lettuce showed the lowest result with the use of R-LEDs alone, and when B_20_R_160_ and B_100_R_80_ were compared, bitterness was found to increase in B_100_R_80_, indicating that blue light increases bitterness [54]. This suggests the possibility that biosynthetic enzymes may be affected by the light quality during the biosynthesis of BSLs. However, there are currently very few studies that clearly report the degree of bitterness and the BSL content of lettuce according to various light qualities. In the case of previous studies on lettuce sourness, a comparison experiment was conducted as part of the sourness sensory evaluation according to the lettuce varieties [56], and an analysis was carried out on the content of acetic acid and lactic acid representing the sour taste in the packaging during the storage of fresh lettuce [57]. However, when compared with other important parameters, sourness, among the taste components in lettuce, was not considered very important, and there was no clear difference; therefore, it was difficult to find accurate information on comparative sourness studies of single varieties of lettuce according to various light qualities. In this study, flavor averaged around 2.5 points, with no significant difference between the treatment groups. Flavor and overall preference also showed no significant differences according to the light quality of the artificial light source [54]. In this study, the texture of lettuce grown under R-LEDs received the best evaluation (Figure 3), and Meng et al. [54] also reported that the highest score was obtained for lettuce grown under R-LEDs alone, followed by BR-LEDs and W-LEDs. In the literature, among the lettuce sensory evaluations according to the use of various LEDs, most of the results of food taste surveys were analyzed for correlations between parameters or the statistical significance of the results until recently, and the biochemical content was rarely mentioned. In addition, content sensory evaluations of lettuce grown under FR-LEDs are rare, and thus, further research should be conducted.

In this study, there was no significant correlation between Hunter a* and ARI1 values, with 0.785 (Figure 4), but this value was similar to the value found in a previous study showing a significant positive correlation, with 0.80 [34]. In addition, ARI1 showed a high positive correlation of 99% with bitterness (Figure 4), which can be attributed to the role of anthocyanins in the bitter taste as the leaf color becomes red. These results suggest that the changes in the anthocyanin content were directly related to bitterness. In a previous study, it was reported that malvidin-3-glucoside, which is an anthocyanin, activates the TAS2R7 receptor among the bitter taste receptors (TAS2R) in humans, resulting in a bitter taste [58]. In addition, it was shown that the light quality that increases the BSL content and the light quality that increases the anthocyanin content may be in the same wavelength range, and thus, additional research is needed. So far, studies on the bitter taste of lettuce have been limited to studies on the difference in the BSL content according to leaf color and cultivar [4], and there are no studies related to light factors, such as the light intensity, photoperiod, and light quality. Therefore, although this study did not investigate the BSL content, it was the first time the degree of bitterness of single cultivar red romaine lettuce according to various light qualities and the relationship between bitterness and anthocyanin content were mentioned. In a previous paper, the total BSL content of red lettuce was significantly higher than that of the green variety, but there was no consistent trend between the total BSL content and sugar content (°Brix) according to the leaf color [4].

## 4. Materials and Methods

### 4.1. Plant Material and Growing Conditions

Mini red romaine lettuce (cv. Breen, Johnny’s Selected Seeds) was used as the material for testing. The temperature and humidity of the closed plant factory-type lettuce cultivation room were controlled at 20 ± 3 °C and 70 ± 5%RH, and the internal CO_2_ concentration was 577 ± 67 ppm without any additional control. Lettuce was planted on a floating platform (50 × 350 × 490 mm) with 40 holes (5 × 8) of 33 mm diameter at a planting interval of 30 mm in a growing tray (130 × 400 × 540 mm). It was cultivated for a total of 49 days (7 weeks) at 7-day intervals using a deep-water culture method in which oxygen was supplied to the water using an aeration pump (SH-A2, Amazonpet, Daejeon, Republic of Korea). The experiment was conducted once, and the above hydroponic cultivation system was installed on a 3-tier shelf for growing. On day 0, 40 individuals were planted under the LEDs for each light quality, and survey items were investigated using 6 individuals on the 14th, 21st, and 28th days; 7 individuals on the 35th and 42nd days; and 8 individuals on the 49th day. As the investigation progressed, empty holes formed by the used lettuce were blocked with a sponge to prevent light from entering the nutrient solution, and the planting distance was increased by using the empty hole to move the seat as the lettuce grew.

### 4.2. Nutrition

The nutrition regime included Yamazaki lettuce nutrient solution and was divided into nutrition formulations A and B. Nutrition formulation A was composed of 472 mg·L^−1^ Ca(NO_3_)_2_·4H_2_O, 404 mg·L^−1^ KNO_3_, and 48 mg·L^−1^ EDTA-NaFe(Ⅲ). Nutrition formulation B was composed of 404 mg L^−1^ KNO_3_, 115 mg L^−1^ NH_4_H_2_PO_4_, 246 mg L^−1^ MgSO_4_·7H_2_O, 6 mg L^−1^ H_3_BO_3_, 4 mg L^−1^ MnSO_4_·5H_2_O, 0.4 mg L^−1^ ZnSO_4_·7H_2_O, 0.1 mg·L^−1^ CuSO_4_·5H_2_O, and 0.04 mg·L^−1^ NO_2_MoO_4_·2H_2_O. Nutrition formulations A and B prepared with the above compositions were used after being diluted 200 times. The pH was set to 6.0 ± 0.5, and the EC was supplied at 0.3 dS·cm^−1^ from the emergence of true leaves and 1.5 dS·cm^−1^ at the end of cultivation.

### 4.3. Light Treatments

**Figure 5:**
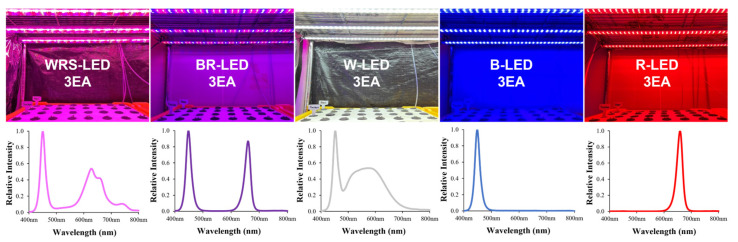
The cultivation environments of LEDs with various light qualities and the spectra of the WRS-LEDs, blue + red-LEDs, white-LEDs, blue-LEDs, and red-LEDs used in the experiment.

**Figure 6:**
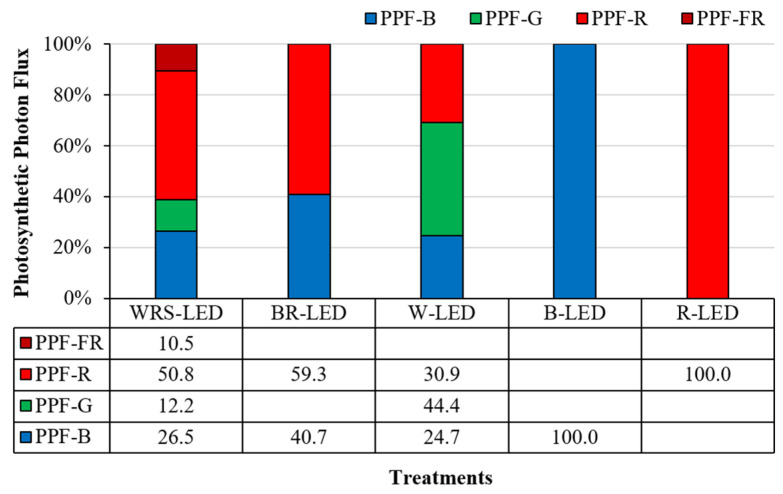
PPF-B (400~499 nm), PPF-G (500~599 nm), PPF-R (600~699 nm), and PPF-NIR (700–780 nm) 100% stacked bar graph for the LED fixtures used in the experiment.

The light-emitting diodes (LEDs) (40 W) were bar-type (20 × 30 × 1200 mm) wide red spectrum (WRS)-LEDs (400–800 nm) (Cheorwon Plasma Research Institute, Gangwon-do, Republic of Korea), blue + red (BR)-LEDs (400–500 nm + 600–700 nm) (HT402-1; BISSOL LED, Seoul, Republic of Korea), white (W)-LEDs (400–700 nm) (HT400-5700; BISSOL LED, Seoul, Republic of Korea), blue (B)-LEDs (400–500 nm) (HT400-Blue; BISSOL LED, Seoul, Republic of Korea), and red (R)-LEDs (600–700 nm) (HT400-Red; BISSOL LED, Seoul, Republic of Korea) (Figure 5 and Figure 6). Each artificial light source was installed on a shelf on the 3rd floor, with 3 per floor, where the LED installation interval was 15 cm and the distance between the LEDs and the floating platform was 25 cm. As the growth progressed, the light intensity was adjusted with a light intensity controller (LED dimmer 20A; ZERO, Daejeon, Republic of Korea) and set to 200 ± 50 μmol·m^−2^·s^−1^ using a Quantum radiometric probe (LP471PAR, Delta OHM, Veneto, Italy) in the dark condition, and the light and dark cycle was set to 16/8 h.

### 4.4. Change in Growth

The length of leaves and the number of leaves were measured every 7 days from the 14th day after planting using 6–8 plants, and the leaf length was measured using an electronic vernier caliper, while the leaf number was counted directly. As for the change in growth at the end of cultivation, the top fresh weight and top dry matter ratio were investigated using 8 plants. The top fresh weight was measured using an electronic scale (PB602-S, Mettler Toledo, Switzerland), and the top dry matter ratio was calculated using the formula shown below after drying at 80 ℃ for 72 h:

Top dry matter ratio = (Dry weight/Fresh weight) × 100

### 4.5. Change in Quality

The leaf color (Hunter L*, a*, b*), soil plant analysis development (SPAD), normalized vegetation index (NDVI), anthocyanin reflectance index 1 (ARI1), and ascorbic acid content were investigated using 8 plants at the end of cultivation. The leaf color (Hunter L*, a*, b*) was measured with a Chroma Meter (CR-400, Konica Minolta Sensing, Inc., Japan), and the chlorophyll content was measured using a chlorophyll meter (SPAD-502 plus, Konica Minolta, Japan). The NDVI and ARI1 were measured with a polypen RP410 (Photon System Instruments Ltd., Drásov, Czech Republic), and the results were calculated according to the following equation:

NDVI = (NIR − Red)/(NIR + Red)

ARI1 = (R550)^−1^ − (R700)^−1^

The ascorbic acid content was determined according to the method of Arvanitoyannis et al. [59]. An amount of 18 mL of distilled water was added to 2 g of the sample, homogenized for 90 s with a homogenizer (HZ1, LABTron Co., Ltd., Bucheon, Republic of Korea), and then centrifuged with a centrifugal separator (Mega 17R, Hanil Science Industrial Co., Incheon, Republic of Korea). Using the supernatant obtained after centrifugation, the ascorbic acid content was measured with an RQ flex reflectometer (Merck RQ flex 2, Darmstadt, Germany).

### 4.6. Sensory Evaluation

Sensory evaluation according to the light quality of the artificial light sources was performed according to the quantitative descriptive analysis (QDA) method, as outlined in the procedures included in the standard Sensory Profiling ISO 13299:2016 [60] used in Matysiak et al. [52]. For the sensory evaluation, on the date of cultivation completion, 15 judges who had experience in the sensory evaluation of vegetables were surveyed on the sugar content, bitterness, sourness, flavor, leaf color, texture, and overall preference. The sensory evaluation score was set in units of 1 point, ranging from 1 to 5 points. Sweetness, bitterness, sourness, and flavor were given a score closer to 5 points if the specific taste and aroma were stronger. As for the leaf color, as the object of study was red romaine lettuce, the deeper the red color, the more points were given. Texture and overall preference were evaluated according to the subjective tendencies of the judges, where the higher the satisfaction, the more points were given.

### 4.7. Statistical Analysis

For statistical analysis, data statistical characteristics (correlation analysis, principal component analysis) were confirmed using the Microsoft Excel 2016 program and IBM SPSS Statistics version 26 program. The data were evaluated via ANOVA (analysis of variance), and the comparison of differences between the averages of the investigation items of the treatment groups was analyzed at the *p* < 0.05 level using Duncan’s multiple range test. The standard deviation (SD) of each mean is indicated.

## 5. Conclusions

Summarizing the above results, the R-LED treatment was the best in terms of growth (leaf length, number of leaves, top fresh weight) according to the LEDs with various light qualities, but it was unsuitable since although it was red lettuce, the leaf color was not red, and the chlorophyll content, NDVI, and ascorbic acid content were the lowest. Conversely, the B-LED treatment produced good quality lettuce but with very low growth; therefore, it was not suitable for lettuce cultivation. WRS-LED was expected to show the best growth and quality change compared with other LEDs used in this study due to its wide light distribution angle, wide red spectrum, and FR light. However, compared with BR-LED, which received a positive evaluation in terms of growth and quality among existing LEDs with various light qualities, there was no noticeable advantage other than equal cultivation within the same treatment group. In this study, the BR-LED treatment produced a great top fresh weight, which is considered important in lettuce cultivation, along with the R-LED treatment. In addition, the value of Hunter a*, which is a measure of leaf redness, and ARI1, which reflects the anthocyanin content, were the highest after the B-LED treatment. The top dry weight ratio, SPAD, and overall preference showed the highest results among all the treatment groups, and ascorbic acid, which acts as an antioxidant, also had the second-highest content. Therefore, the BR-LED treatment was the most suitable for growing mini red romaine lettuce (cv. Breen) in a closed chamber.

## Figures and Tables

**Table 1:** Top fresh weight and top dry matter ratio of mini red romaine lettuce cultivated under LEDs with various light qualities on the final day.

Treatments	Top Fresh Weight (g)	Top Dry Matter Ratio (%)
WRS-LED	72.41 ± 2.97 b^z^	5.21 ± 0.33 a
BR-LED	74.47 ± 3.10 b	5.48 ± 0.39 a
W-LED	57.61 ± 4.18 c	5.03 ± 0.33 ab
B-LED	56.65 ± 5.77 c	5.35 ± 0.20 a
R-LED	100.18 ± 9.92 a	4.68 ± 0.22 b

^z^ Means with different letters within column indicate statistically significant differences by Duncan’s multiple range test at the 5% level.

**Table 2:** Leaf color of mini red romaine lettuce cultivated under LEDs with various light qualities on the final day.

Treatments	Hunter L*	Hunter a*	Hunter b*
WRS-LED	35.53 ± 2.06 c^z^	2.20 ± 1.32 b	9.68 ± 2.72 b
BR-LED	32.48 ± 0.80 d	4.37 ± 0.34 a	3.03 ± 1.23 c
W-LED	38.25 ± 1.37 b	1.12 ± 1.02 c	9.50 ± 2.47 b
B-LED	32.27 ± 0.48 d	4.92 ± 0.30 a	3.20 ± 0.58 c
R-LED	47.83 ± 1.40 a	−4.43 ± 0.54 d	26.93 ± 2.93 a

^z^ Means with different letters within column indicate statistically significant differences by Duncan’s multiple range test at the 5% level.

**Table 3:** SPAD, NDVI, ARI1, and ascorbic acid of mini red romaine lettuce cultivated under LEDs with various light qualities on the final day.

Treatments	SPAD	NDVI	ARI1	Ascorbic Acid (mg/100 g FW)
WRS-LED	31.0 ± 0.52 c^z^	0.519 ± 0.02 b	0.754 ± 0.18 b	4.40 ± 0.23 a
BR-LED	38.8 ± 0.68 a	0.530 ± 0.01 ab	0.809 ± 0.29 b	4.15 ± 0.73 a
W-LED	29.5 ± 0.93 d	0.471 ± 0.03 c	0.473 ± 0.03 b	3.39 ± 0.17 b
B-LED	34.9 ± 0.47 b	0.550 ± 0.02 a	1.951 ± 0.52 a	4.03 ± 0.14 a
R-LED	29.3 ± 2.16 d	0.441 ± 0.03 d	0.125 ± 0.03 c	2.73 ± 0.38 c

^z^ Means with different letters within column indicate statistically significant differences by Duncan’s multiple range test at the 5% level.

## Data Availability

Data is contained within the article.
